# Aetiology of Dental Caries

**Published:** 1907-10

**Authors:** T. M. Allen

**Affiliations:** Tampa, Fla.


### AETIOLOGY OF DENTAL CARIES.

By T. M. Allen, D. D. S., Tampa, Fla.

Aetiology is that branch of science which treats of the cause of diseases,

Diseases are classified, aetiologically into idiopathic and symptomatic. The former or idiopathic class are such as cannot be traced to any known cause. The latter or symptomatic are such as can be traced by symptoms or otherwise to a cause.

There are two divisions or causes of diseases, predisposing and exciting, by predisposing causes, or any pre-exciting bodily conditions functional habitude, pecularity of internal structure, or external form, which creates a tendency to disease, or renders favorable its development. Predisposing causes should be studied very carefully and minutely. By this means we can very often ascertain the cause, and by knowing the cause of a disease, we are better able to treat it, and very often can remove the cause and make a successful cure easily and quickly, when without this knowledge would require a long and tedious course of treatment and may finally result in a failure to cure. And some other practitioner not nearly so well qualified as you are, may take the case, and being a close observer ascertain the cause and by this knowledge make a success when you have made a failure.

There is too little importance attached to the predisposing cause of disease and especially of dental decay. A more careful study and knowledge of the predisposing causes of dental decay with a proper knowledge of Pathology and Therapeutics will enable you to successfully treat and arrest this disease.

Exciting causes are those which immediately precede the development of disease and operate to produce it.

The greatest and almost universal cause of dental disease, is the wasting or destruction of tooth substance by decay.

The most noticeable feature of dental decay or caries, first:—Is a solution of the enamel at a point on the tooth least exposed to friction or favorable for the retention of foreign substances. Second:—A solution of the mineral portion of the dentine at the bottom of the cavity by the wasting away of the enamel at the point. Third:—A dissolving or crumbling out of the animal or organic part of the dentine.

The natural causes or agencies that operate to disintegrate enamel and dentine and other lime formation are slowly disintegrated by atmospheric agencies, vapors, etc.

The artificial agencies that are the most destructive and greatly accelerate decay and disease of the tooth structure are acids. Both of these agencies are present and operate in the mouth. The atmosphere by inhalation; and the acids are produced by chemical decomposition of food remaining between or about the necks of the teeth, or resulting from a condition of the stomach or salivary and mucous glands. By the decomposition of vegetable substance in the mouth, acetic acid is formed, and by gastric and glandular disturbances, hydrochloric and lactic acids are formed.

Chemistry teaches us that these acids are most active in a nascent state, thus wasting their strength in the immediate locality where they are formed. You must not think that by the mere fact of an acid condition of the fluids of the mouth, that they can not be considered as a prime factor in dental decay.

There are other and more potent causes of dental decay than the acid condition of the saliva. Organic tissue both animal and vegetable are destroyed by the combined action of the elements every where prevalent in nature, viz.: heat moisture and oxygen. These elements are always present in the mouth, heat from the animal heat of the body, moisture from the saliva, oxygen from the inhalation of the atmosphere. Either one of the three being absent the destruction of the tissue ceases.

This is easily demonstrated by the canning process of meats, vegetables, and fruits by which the air is excluded, by the process of desiccation of the same to exclude moisture; by freezing to exclude heat, and as long as they remain in this condition, there is no fermentation and consequently no decay.

The principle of filling teeth as a prophylactic treatment of decay, is based on the principles just stated, the exclusion of the destructive agent, and by filling them the object is to exclude the moisture and air. And when this is successfully done the decay of the tooth is arrested and the tooth will be free from further decay as long as this condition lasts.

The micro-organism theory as in connection with dental decay is that after the penetration of the enamel by the action of acids produced chemically, bacterial germs from the atmosphere or other sources enter the cavity and there germinate and propogate in great numbers; that they exclude an acid poison at once destructive to a limited depth of both the vitality of the dentine and mineral elements composing it; that by crowding into the tubule they break down the intervening walls of the tubules, that the animal portion thus becoming freed from its combinations, is devoured by the bacteria as sustenance.

This, mind you, is mere theory and the facts have not been demonstrated yet. The presence of bacteria in all waste or decaying substances has been demonstrated, but their relations to and the part they bear in the destructive processes of dental decay is not known.

Their presence and functional activities may well be disposed to promote decay by a continual irritation rendering it necessary in the treatment of decay to employ a germicidal agent. Some of the predisposing causes of decay, inherited types, tendency and functional action in development of the tissues inherited taints of constitutional disease, faulty nutrition at the time of development by reason of which the relative proportions of organic or inorganic material needed for an enduring structure fail to be supplied; faulty form manifested by a rough indented surface, prominent cusps and deep intervening tissues on the crowns of bicuspids and molars; broad grinding surfaces in contact and broad triangular apices intervening at necks of the teeth which furnish pockets for foreign matter; and all gastric disturbances or other diseased conditions of the body that create a viciated condition of the fluids of the mouth.

Of the predisposing causes, the inherited idiosynciosies probably influence the development of dental decay in the largest number of cases. The parent is the complete type of the child and is the medium of transmitting type from a preceding generation to every tissue and function of the body, and the teeth are not exceptions to this rule.

The proper prophylactic treatment of dental decay, is the securing of the general health of the body and absolute cleanliness of the teeth and mouth to prevent the introduction of disease, and the operation of filling the teeth to arrest its progress. And when it has already commenced the vitality of the teeth renders considerable resistance to the progress of decay. If the progress of decay is slow, there is resistance afforded by filling up of the dentinal tubules with a calcarious formation called secondary dentine, thus increasing the density of the tooth structure, and checking the rapidity of the progress of decay. Under some favorable circumstances arresting it entirely, as shown in those hard dense, dark spots we often see in the teeth, and by proper treatment we can often cause a secondary deposit of dentine and arrest the further progress of decay.

In teeth with deep seated cavities with a thin septum of dentine over the pulp, if this be treated by applying a paste made of tannic acid and oil of cloves, placing this over the pulp covering the semi-decomposed dentine and covering this with cement, we will find that a deposit of secondary dentine will be thrown off rendering the soft semi-decomposed dentine over the pulp, and in the bottom of the cavity covered by the tannic acid and oil of cloves of a hard dense horny consistency. Much more dense than the dentine of the other portion of the tooth and not near so sensitive or susceptible to decay. Showing that by proper treatment we can improve the tooth substance and increase their density and resistance to decay. And especially do we find this the case with children's and young people's teeth.

